# Intraoral electrical muscle stimulation in the treatment of snoring

**DOI:** 10.1007/s11818-018-0179-z

**Published:** 2018-09-06

**Authors:** E. Wessolleck, E. Bernd, S. Dockter, S. Lang, A. Sama, B. A. Stuck

**Affiliations:** 1 Department of Otorhinolaryngology, Head and Neck Surgery, University Hospital Marburg, Philipps-Universität Marburg, Baldingerstraße, 35043 Marburg, Germany; 2 Department of Otorhinolaryngology, Head and Neck Surgery, University Hospital Essen, University Duisburg-Essen, Essen, Germany; 3 Section of Phoniatrics and Pediatric Audiology, Department of Otorhinolaryngology, Head and Neck Surgery, University Hospital Essen, University Duisburg-Essen, Essen, Germany; 4 Nottingham University Hospital, Nottingham, UK

**Keywords:** Pharyngeal muscles, Sleep related breathing disorder, Electric stimulation therapy, Sleep apnea syndrome, Muscle tonus, Pharyngeale Muskulatur, Schlafbezogene Atmungsstörung, Muskelstimulation, Schlafapnoe, Muskeltonus

## Abstract

The tone of the intraoral und pharyngeal muscles of the upper airway is of particular importance for the development of snoring. By increasing the tone with electrical stimulation, a reduction in snoring may be achieved. The aim of the study was to record the effects of intraoral muscle stimulation during the day on snoring at night.

The prospective bi-centric study included 16 patients with snoring and mild obstructive sleep apnoea (Apnoea Hypopnoea Index [AHI] < 15, BMI < 32). After initial polygraphy, snoring was monitored over 2 weeks (baseline) using a visual analogue scale (VAS; 0–10). This was followed by a 6-week treatment phase (2 × 20 min daily) with an intraoral electrical stimulation device. During and up to 2 weeks after therapy, snoring intensity in addition to use and potential side effects were documented on a daily basis.

Three patients discontinued therapy because of technical problems. The 13 remaining patients (11 male/2 female, BMI 26.9 ± 3.2, AHI 9.3 ± 4.6) underwent per-protocol analysis. The mean snoring score was reduced from 5.6 ± 1.1 (baseline) to 3.2 ± 2.7 (after therapy) and remained stable until 2 weeks after treatment (3.3 ± 2.4). In 7 patients (53.9%) the score was reduced by more than 50%. Patients with an AHI < 10 responded better to therapy. No unexpected events occurred.

In the present pilot study, the first signs of the effectiveness of intraoral muscle stimulation in snoring patients were shown. In addition to a technical improvement of the stimulator, carrying out controlled trials and assessing potential influencing factors on the success of therapy are necessary.

## Zusammenfassung

Der Tonus der intraoralen und pharyngealen Muskulatur des oberen Atemweges ist für die Entstehung des Schnarchens von besonderer Bedeutung. Durch eine Tonuserhöhung über eine elektrische Stimulation könnte eine Reduktion des Schnarchens erreicht werden. Ziel der Studie war, die Effekte einer intraoralen Muskelstimulation am Tag auf das Schnarchen in der Nacht zu erfassen.

In die prospektive bizentrische Studie wurden 16 Patienten mit Schnarchen und leichtgradiger Schlafapnoe (Apnoe-Hypopnoe-Index [AHI] < 15, Body Mass Index [BMI] < 32) eingeschlossen. Nach initialer Polygraphie wurde das Schnarchen über zwei Wochen (Baseline) mittels visueller Analogskala (VAS; 0–10) erfasst. Hieran schloss sich eine sechswöchige Therapiephase (täglich 2 × 20 min) mit einem intraoralen elektrischen Stimulationsgerät an. Während und bis zwei Wochen nach der Therapie erfolgte eine tägliche Dokumentation der Schnarchintensität sowie der Nutzung und möglicher Nebenwirkungen.

Drei Patienten brachen die Therapie aufgrund technischer Probleme mit dem Gerät ab. Bei den 13 verbliebenen Patienten (11m/2w, BMI 26,9 ± 3,2, AHI 9,3 ± 4,6) erfolgte eine „per-protocol“ Analyse. Der gemittelte Schnarchscore wurde von 5,6 ± 1,1 (Baseline) auf 3,2 ± 2,7 (nach Therapie) reduziert und erwies sich bis zwei Wochen nach Therapie als stabil (3,3 ± 2,4). Bei sieben Patienten (53,9 %) zeigte sich eine Reduktion des Scores um über 50 %. Patienten mit einem AHI unter 10 sprachen besser auf die Therapie an. Unerwünschte Wirkungen traten nicht auf.

In der vorliegenden Pilotstudie ergaben sich erste Hinweise auf die Wirksamkeit einer intraoralen Muskelstimulation bei Patienten mit Schnarchen. Notwendig sind neben einer technischen Verbesserung des Stimulators die Durchführung kontrollierter Studien sowie die Untersuchung möglicher Einflussfaktoren auf den Therapieerfolg.

The reduction of muscle tone during sleep leads to a reduction in airway diameter, and therefore to a noticeable increase in airflow velocity during inspiration—and occasionally also during expiration. Above a critical speed threshold, local pressure variations occur at specific bottlenecks in the upper aero-digestive tract. Consequently, these lead to vibrations of the soft tissue and their resulting sounds. The most relevant anatomical structures that contribute to this are the palatal arch, the sides of the pharynx including the adenoids, the base of the tongue, and the epiglottis.

Snoring can therefore by classified as a commonly inspiratory but also expiratory sound associated with sleep, and takes place in the upper airway. Snoring is considered “disruptive” (cf. “primary” or “habitual”) if it occurs without quantitative evidence of airflow limitations (e. g. apnoea or hypopnoea) [14].

The prevalence of snoring is especially pronounced in middle age. In a standardised telephone interview, 62% of men and 45% of women aged between 45 and 54 years stated that they regularly snore [9].

A multifactorial aetiology can be assumed. Important risk factors for the development of snoring have been identified to be nasal obstruction, male sex, age and excess weight [2, 6, 8]. Moreover, there is a direct association with alcohol and nicotine consumption.

Unlike obstructive sleep apnea (OSA), disruptive snoring is not considered a disease with medical danger to the affected person, according to the current state of scientific knowledge. For this reason, there is no need for medical treatment, according to latest expert opinion [14].

Nonetheless, subjects who snore present with relevant complaints. It is typical that the quality of life of the bedpartner, specifically, is reduced. Women with partners who snore often complain about sleep disorders, headaches in the morning, and fatigue during the day [15]. According to a study published in 2005, 55% of bedpartners of patients with sleep-related breathing disorders are disturbed almost every night, 40% sleep in another room at least once a week, 26% regularly use earplugs or sleeping pills, and 35% report at least intermittent relationship problems occurring due to the snoring [17].

The snorers themselves also show evidence of a reduced quality of life. There are signs of an association of snoring with the development of hoarseness, headaches, scary dreams, and poor sleep quality [3, 5, 12].

In summary, disruptive snoring is a phenomenon without direct impact on the morbidity and mortality of patients; however, it has significant potential to induce a high level of suffering for both the snorer and their personal and/or domestic environment, which quite often leads them to resort to medicinal services.

Numerous treatment options are available to treat disruptive snoring [14]. Amongst these, especially general measures like weight loss and alcohol and/or nicotine abstention are noteworthy. Possible instrument-based therapies are vests to prevent the supine position during sleep, diverse sleep position trainers, and mandibular advancement devices. When it comes to surgical treatment options, they primarily concern interventions at the soft palate and in the nasal septum and conchae. Thereby, one must bear in mind that there is little scientific data available to support general measures and positional treatment for snoring [14], and not all snorers prove suitable for mandibular advancement devices or surgical procedures. In addition, the short- and long-term consequences of these measures should be considered, particularly given that they are not medically necessary.

Furthermore, surgical procedures are not sustainably effective in all cases [14], and the treatment costs are usually not covered by health insurance. As a result, there is a demand for a non-invasive, affordable alternative treatment method.

## Muscle stimulation to treat snoring

In two independent studies on the effectiveness of electrical muscle stimulation in OSA, it has already been shown that despite not bringing about a significant reduction of the Apnoea–Hypopnoea Index (AHI), it did lead to an improvement in snoring. The number of snoring periods within the training groups significantly decreased [11, 16]. Due to the lack of effectiveness in OSA, this alternative treatment was not pursued, and at present there are no data on its application to disruptive snoring.

Given this background, a CE-certificated stimulation device (SnooZeal©, Snoozeal Limited, London, GB) was developed. It lies completely intraorally, and has been especially designed to treat patients with disruptive snoring. The mouthpiece is placed under the tongue, and the electrodes for muscle stimulations over the tongue, through which a pulsing stimulation occurs within set parameters.

Risk of electric or thermal damage is not expected with the present licensing parameters, given pre-defined device settings and their limited adjustability by patients.

## Aims of the study

In this study, the hypothesis was put forward that electrical stimulation of the intraoral musculature with the newly developed intraoral stimulator improves snoring in the absence of OSA or presence of mild OSA.

## Materials and methods

### Methods

In the bicentric prospective study (Department of Otorhinolaryngology, Head and Neck Surgery, University Hospital Essen, Germany; Nottingham University Hospital, Nottingham, United Kingdom), a total of 16 patients (body mass index [BMI] < 32) with snoring and mild sleep apnea (AHI < 15) were tested.

Initially, a medical history, ENT examination and out of center sleep testing was performed to exclude relevant sleep-related breathing disorders. The patients snored regularly for at least the last 6 months and did not suffer from an OSA requiring treatment (AHI < 15). Additionally, no serious nasal pathologies, e. g. a pronounced septal deviation, should exist in the patient set. A further critical inclusion criterion was the existence of a bedpartner who documented the snoring intensity and changes during and after the treatment period. A comprehensive list of the inclusion and exclusion criteria for participation in the study is provided in Tables 1 and 2.

**Table 1:** Inclusion criteria for participation in the study

Age: 20–65 years
Sex: male and female
History of more than 6 months’ continuous snoring (>5 times per week)
Bedpartner to document snoring intensity must be available

**Table 2:** Exclusion criteria for participation in the study

Epworth Sleepiness Score (questionnaire on self-assessment of daytime fatigue) > 10
Body mass index (BMI) > 32 kg/m^2^
AHI (Apnea–Hypopnea Index) > 15, and therefore proof of an OSA in outpatient recording or polysomnography, not older than 6 months before inclusion
Symptomatic nasal pathology: septum deviation, nasal polyposis or chronic rhinosinusitis with corresponding nasal obstruction
Tonsil hyperplasia (tonsil size > grade 2 or 25–50% obstruction of the upper airway)
Tongue or lip piercing
Pacemaker or implanted medical electrical devices
Previous surgical treatment in the oropharyngeal area for a sleep-related breathing disorder
Relevant deformity of the facial skeleton (e. g. severe micrognathia, midface hypoplasia)
History of pregnancy

The training frequency and duration was 2 × 20 min daily. During the treatment phase and up to 2 weeks after the end of the treatment (post-treatment phase, day 43–56), the snoring intensity was documented daily by the bedpartner according to a visual analogue scale (VAS: 0–10; subsequently referred to as ‘snoring score’), as well as potential side effects, which were documented by the patient.

Data collection was carried out anonymously and according to the current data protection guidelines. The required devices were provided by the manufacturers free of charge, and remained in the possession of the subjects after completion of the study. The clinic received remuneration of expenses from the sponsor of the study or manufacturer, respectively. Patients could leave the study at any time without providing a reason. After appropriate explanation, the subjects provided their written consent to take part in the study. The study protocol was examined by the responsible ethics commissions, and its implementation was in accordance with the provisions laid out in the Declaration of Helsinki.

**Fig. 1:**
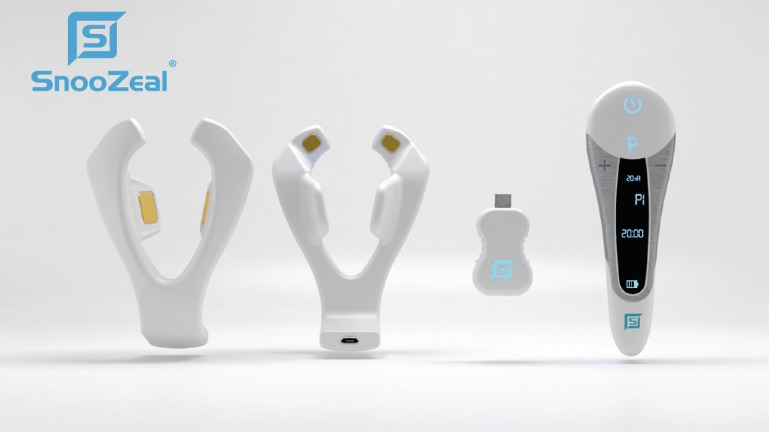
Illustration of the SnooZeal© device (Snoozeal Limited, London, GB). Two images on the left show the intraoral device element from the front and back, two images on the right show the control unit and remote control respectively. Reproduced with permission of the manufacturer

Upon inclusion in the study, the snoring intensity was recorded via VAS by the bedpartner for 2 weeks (baseline: day −14 to −1). Only thereafter did the six-week treatment phase (day 1–42) commence with the intraoral simulation device (Fig. 1). The evaluation of the effectiveness of the treatment is based on averages of the three treatment periods: pre-treatment (2 weeks), the treatment phase (the last 2 weeks) and post-treatment (2 weeks). For this, the mean snoring scores of the first 2 observation weeks (pre-treatment score week 1 and 2), the last treatment weeks (treatment score weeks 5 and 6), and the 2 post-treatment weeks (post-treatment score week 1 and 2) were used.

### Statistics

The data were tested using the Kolmogorov–Smirnow test for normal distribution (not normally distributed). Comparison of the values before and after the treatment was conducted using the Wilcoxon rang sum test.

## Results

In total, 16 patients were included in the study. Three patients left the study due to technical problems with the device during the treatment.

Of the 13 remaining patients, 11 were male and 2 were female. The oldest patient was 59 years old, the youngest 25. On average, the patients’ age was 43.2 ± 12.2 years. The BMI was on average 26.9 ± 3.2 kg/m^2^. In the study population, the mean AHI was 9.3 ± 4.6. The mean score of the Epworth Sleepiness Scale (ESS) was 7.4 ± 2.3. An overview of the epidemiological data of the research population is provided in Table 3.

**Table 3:** Overview of the epidemiological data of the study

No	Sex	Age	BMI	ESS	AHI
1	M	35	27.9	9	12.5
2	M	52	31.1	5	3.5
3	M	36	31.2	10	4.8
4	F	25	20.7	4	5.5
5	M	59	24.6	8	4.4
6	M	28	26	7	2.5
7	F	59	22.6	10	13.1
8	M	56	26	10	13.4
9	M	38	26.6	10	13.5
10	M	28	25.5	5	11.5
11	M	49	30.1	7	14.1
12	M	49	29.1	4	13.9
13	M	48	28.4	7	7.7
Average		43.2	26.9	7.4	9.3
Median		48	26.6	7.0	11.5
Standard deviation		12.2	3.2	2.3	4.6

*BMI* Body Mass Index, *ESS* Epworth Sleepiness Scale, *AHI* Apnea–Hypopnea Index, *M* male, *F* female

**Fig. 2:**
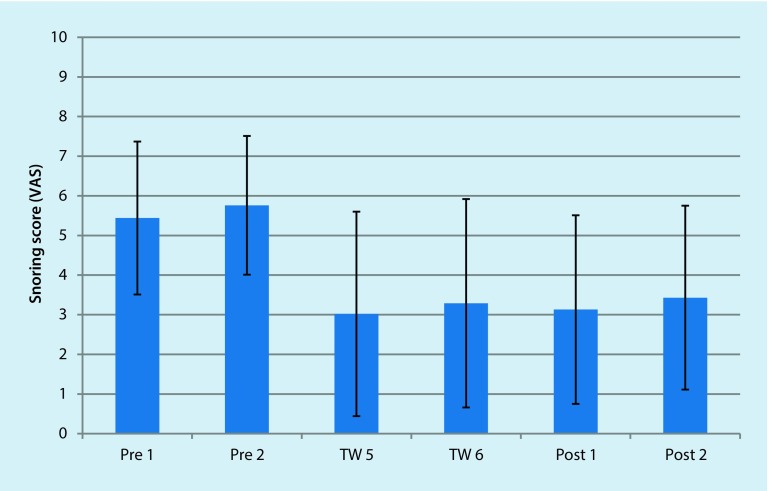
Graphical representation of the change in snoring VAS scores of all patients over the treatment period (average and standard deviation). *Pre* pre-treatment score week 1 and 2, *TW* treatment week scores week 5 and 6, *Post *post-treatment score week 1 and 2 post completion of treatment

The average snoring score was statistically significantly (*p* < 0.05) reduced from 5.6 ± 1.1 (baseline) to 3.2 ± 2.7 (after treatment), and remained stable up to 2 weeks after the treatment (3.3 ± 2.4). The snoring score was lowered on average by 44% by the end of the treatment phase. This effect was not weakened even 2 weeks after the end of the treatment (with an average reduction of the snoring score by 43%). With 7 patients (53.9%), a reduction of the snoring score of above 50% was noted. An overview of the snoring scores over time is shown in Fig. 2 and Table 4.

**Table 4:** Overview of the results of the study with the formation of 2 groups (AHI below 10/h and AHI 10 or above)

No	AHI	Score week 1 (Pre 1)	Score week 2 (Pre 2)	Mean Pre 1 + 2	Score treatment week 5 (TW 5)	Score treatment week 6 (TW 6)	Mean TW 5 + 6	Score after treatment week 1 (Post 1)	Score after treatment week 2 (Post 2)	Mean Post 1 + 2
2	3.5	4.54	4.72	5.65	1.07	0.99	1.03	0.89	1.05	0.97
3	4.8	6.05	7.27	4.63	3.26	3.17	3.22	3.66	3.21	3.44
4	5.5	2.19	3.02	3.57	0.98	0.78	0.88	1.12	0.85	0.99
5	4.4	4.96	4.11	5.14	0.33	1.15	0.74	0.65	2.1	1.38
6	2.5	5.65	5.83	6.91	1.29	3.71	2.50	2.56	3.15	2.86
13	7.7	7.46	8.71	6.73	0.31	0.43	0.37	0.49	0.13	0.31
Mean	4.73	5.14	5.61	5.44	1.21	1.71	1.46	1.56	1.75	1.66
SD	1.63	1.61	1.92	1.16	0.99	1.26	1.03	1.16	1.16	1.11
1	12.5	5.94	5.65	5.80	3.07	3.23	3.15	3.15	3.52	3.34
7	13.1	7.10	6.75	4.66	1.97	1.41	1.69	1.24	2.16	1.70
8	13.4	1.37	3.40	5.20	0.67	0.77	0.72	1.96	2.74	2.35
9	13.5	7.79	8.24	6.52	7.79	8.27	8.03	7.41	8.03	7.72
10	11.5	5.04	5.00	4.84	5.77	5.03	5.40	4.36	5.21	4.79
11	14.1	4.59	4.73	6.20	5.56	6.17	5.87	5.69	5.12	5.41
12	13.9	7.99	7.50	7.75	7.19	7.69	7.44	7.56	7.38	7.47
Mean	13.14	5.69	5.90	5.85	4.57	4.65	4.61	4.48	4.88	4.68
SD	0.77	2.00	1.47	0.94	2.34	2.56	2.44	2.18	1.94	2.05
Mean All	9.26	5.44	5.76	5.66	3.02	3.29	3.16	3.13	3.43	3.28
SD All	4.38	1.93	1.75	1.10	2.58	2.63	2.58	2.38	2.32	2.33

*AHI* Apnea–Hypopnoea Index, *SD* standard deviation, *Pre* pre-treatment score week 1 and 2, *TW* treatment week scores week 5 and 6, *Post* post-treatment score week 1 and 2 post completion of treatment

**Fig. 3:**
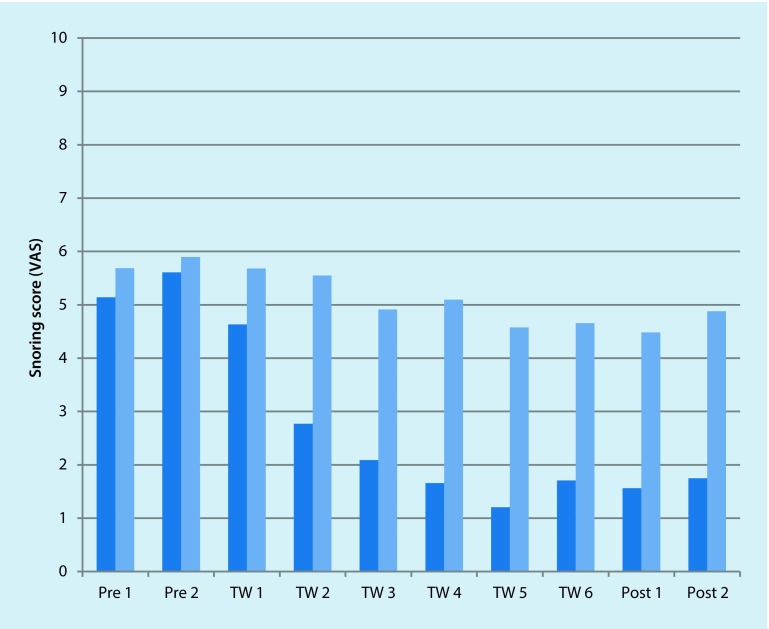
Graphical representation of the change of the snoring scores of patients with an Apnea–Hypopnea Index (AHI) below 10/h (*dark blue columns*) and patients with an AHI 10 and above (*light blue columns*). *Pre* pre-treatment score, *TW* treatment week, *Post* post-treatment score

It was noteworthy that the patients with an AHI below 10 (*n* = 6) could benefit more markedly from the treatment. This relationship is shown in Fig. 3 and Table 4. Moreover, there was a negative correlation between the reduction percentage of the snore score and the AHI.

The patients reported a subjective feeling of electrical stimulation in the mouth during the training phase as a short “twitching” or “tingling”. No undesirable effects arose in the study group.

## Discussion

In various studies, it has already been shown that by training the upper breathing musculature by playing a wind instrument (didgeridoo; [10]) and corresponding oropharyngeal exercises [1, 4, 7], a moderate OSA can be alleviated.

As demonstrated in the current meta-analysis, oropharyngeal exercises can reduce the AHI by 50% in adults, and consequently improve snoring [1]. It is suspected that these effects were caused by a change in oropharyngeal muscle tone. This is supported by observations that transcutaneous electrical stimulation appears to affect a noticeable increase in muscle strength and base muscle tone in paralysed or inactive limbs [13]. As the muscles of the pharynx and tongue, as well as the limbs, are skeletal muscles, an electrical or electromagnetic stimulation of the pharyngeal and tongue musculature can affect an increased muscle tone above the base tone during sleep, and therefore reduce the tendency for the pharynx to collapse and result in snoring.

As illustrated, this principle has already been attempted to be used in treatment for OSA [11, 16]. Randerath et al. [11] included 67 patients with mild to severe OSA (AHI 10–40) in a randomised, placebo-controlled, double-blind study on electrical stimulation of the tongue musculature. The biphasic stimulation took place using an electrode positioned under the tongue, and a further electrode that was attached to the floor of the mouth from the outside. In this study, no effect was noticed on AHI; however, a significant effect on snoring was observed (the snoring episodes were lowered from 63.9 ± 23.1 episodes/h to 4.5 ± 31.2; *p* < 0.05). In the placebo group, this effect was not demonstrated.

A further study by Verse et al. [16] took place with 15 patients with OSA or Upper Airway Resistance Syndrome (UARS). These received transcutaneous electric stimulation via two conventional electrodes attached to the skin (ECG-electrodes), which were positioned submentally. Rather than objective breathing parameters, in this study clear improvements were detected in subjective evaluations of snoring by the bedpartners. On average, a decrease from 7.0 ± 2.2 to 3.4 ± 2.0 points on the VAS was claimed (*p* = 0.005).

Also, in the present pilot study there were indications of effectiveness of intraoral muscle stimulations of patients with snoring, in relation to the bedpartners’ stated subjective snoring intensity.

As already shown in the results section, especially patients with an AHI below 10 (*n* = 6) could benefit from the treatment. These patients’ snoring score decreased on average by 68%. The patients with an AHI of 10 and above (*n* = 7), in contrast, only benefitted minimally from the treatment, although the small sample size of this subpopulation cannot allow for reliable conclusions to be drawn. This is somewhat inconsistent with the results of the aforementioned studies on electrical stimulation of patients with OSA for whom a significant decline in snoring was also established.

## Practical conclusion

- The presented treatment with a newly developed device for intraoral muscle stimulation has the potential to be effective particularly in the treatment of disruptive snoring.
- Further prospective studies with larger groups and a controlled design are in preparation to investigate the value of this procedure more thoroughly.
